# Phenotype‐Based Isolation of Antigen‐Specific CD4^+^ T Cells in Autoimmunity: A Study of Celiac Disease

**DOI:** 10.1002/advs.202104766

**Published:** 2022-02-04

**Authors:** Asbjørn Christophersen, Shiva Dahal‐Koirala, Markéta Chlubnová, Jørgen Jahnsen, Knut E. A. Lundin, Ludvig M. Sollid

**Affiliations:** ^1^ KG Jebsen Coeliac Disease Research Centre University of Oslo Oslo 0372 Norway; ^2^ Institute of Clinical Medicine University of Oslo Oslo 0450 Norway; ^3^ Department of Rheumatology Dermatology and Infectious Diseases Oslo University Hospital Oslo 0372 Norway; ^4^ Department of Gastroenterology Akershus University Hospital Lørenskog 1478 Norway; ^5^ Department of Gastroenterology Oslo University Hospital Rikshospitalet Oslo 0372 Norway; ^6^ Department of Immunology Oslo University Hospital Oslo 0372 Norway

**Keywords:** antigen‐specific T cells, autoimmunity, celiac disease, HLA tetramers, T cells

## Abstract

The pathogenic immune response in celiac disease (CeD) is orchestrated by phenotypically distinct CD4^+^ T cells that recognize gluten epitopes in the context of disease‐associated HLA‐DQ allotypes. Cells with the same distinct phenotype, but with elusive specificities, are increased across multiple autoimmune conditions. Here, whether sorting of T cells based on their distinct phenotype (Tphe cells) yields gluten‐reactive cells in CeD is tested. The method′s efficiency is benchmarked by parallel isolation of gluten‐reactive T cells (Ttet cells), using HLA‐DQ:gluten peptide tetramers. From gut biopsies of 12 untreated HLA‐DQ2.5^+^ CeD patients, Ttet^+^/Tphe^+^, Ttet^−^/Tphe^+^, and Ttet^−^/Tphe^−^ cells are sorted for single‐cell T‐cell receptor (TCR)‐sequencing (*n* = 8) and T‐cell clone (TCC)‐generation (*n* = 5). The generated TCCs are TCR sequenced and tested for their reactivity against deamidated gluten. Gluten‐reactivity is observed in 91.2% of Ttet^+^/Tphe^+^ TCCs, 65.3% of Ttet^−^/Tphe^+^ TCCs and 0% of Ttet^−^/Tphe^−^ TCCs. TCR sequencing reveals clonal expansion and sequence sharing across patients, features reflecting antigen‐driven responses. The feasibility to isolate antigen‐specific CD4^+^ T cells by the sole use of phenotypic markers in CeD outlines a potential avenue for characterizing disease‐driving CD4^+^ T cells in autoimmune conditions.

## Graphical abstract

A distinct CD4^+^ T‐cell phenotype is increased across multiple autoimmune disorders. Here, a proof‐of‐concept is presented for direct isolation of antigen‐specific T cells based on this distinct phenotype of autoimmune conditions using celiac disease as a test case. The majority of isolated cells are specific to gluten, and T‐cell receptor repertoire analysis indicates antigen‐driven responses.

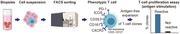

## 1 Introduction

Autoimmune diseases are complex disorders with the involvement of multiple susceptibility genes.^[^ ^1^ ^]^ Across diseases, it is observed that human leuckocyte antigen (HLA) (major histocompability complex, MHC) genes have by far the biggest effect size.^[^ ^2^ ^]^ The HLA association with disease is best explained by the preferential presentation of antigen by the disease‐associated allotypes, yet the disease‐driving antigens remain unknown for the majority of HLA‐associated disorders. For seropositive autoimmune diseases, the primary HLA‐association is with HLA class II alleles suggesting a key role of CD4^+^ T cells. Conceivably, isolation of pathogenic CD4^+^ T cells and insights into their T‐cell receptor (TCR) repertoires may lead to the identification of disease‐driving self and/or foreign antigens, through TCR‐directed approaches with peptide‐MHC libraries and by computation.^[^ ^3^, ^4^, ^5^ ^]^ By identification of pathogenic CD4^+^ T cells in autoimmune disease, novel targets for immunotherapy may be identified.

Among HLA‐associated diseases, celiac disease (CeD) is fairly unique in that much is known about the adaptive immune response, and particularly that pathogenic, disease‐driving CD4^+^ T cells specific for deamidated gluten epitopes have been identified.^[^ ^6^ ^]^ Such gluten‐specific CD4^+^ T cells are present in the gut and blood of CeD patients, but not in healthy controls.^[^ ^6^, ^7^ ^]^ Gluten are proline‐ and glutamine‐rich proteins of wheat (gliadin/glutenin), rye (secalin), and barley (hordein), which can be posttranslationally deamidated by the enzyme transglutaminase 2 (TG2), an enzyme that is the target antigen for autoreactive B cells in CeD patients.^[^ ^8^ ^]^ These deamidated gluten epitopes are presented exclusively by HLA‐DQ2.5 (carried by ≈90% of the patients), HLA‐DQ2.2, or HLA‐DQ8.^[^ ^9^ ^]^ Some of these epitopes elicit responses in most patients, and the CD4^+^ T cells specific for these epitopes dominate the clonal responses; such epitopes are termed immunodominant epitopes.^[^ ^10^ ^]^ To date, >20 gluten epitopes have been characterized, yet many gluten epitopes remain unidentified.^[^ ^11^ ^]^ Of note, the CD4^+^ T cells specific for given epitopes of gluten often use similar TCRs across patients, thus reflecting the existence of TCRs that are shared across patients, so‐called public TCRs, in CeD.^[^ ^12^, ^13^, ^14^ ^]^ Public TCR repertoires are observed in various conditions and are considered to reflect TCRs particularly suited to recognize a given peptide–MHC complex.^[^ ^15^ ^]^

Antigen‐specific CD4^+^ T cells can be isolated directly from blood and tissue with HLA multimers, like HLA tetramers,^[^ ^16^, ^17^ ^]^ or detected by proliferation assays or detection of secreted analytes, using ELISPOT.^[^ ^18^ ^]^ However, all these techniques require prior knowledge about the disease‐driving antigens. An alternative approach, to isolate the antigen‐specific and autoreactive CD4^+^ T cells solely based on their phenotype, would require knowledge about the phenotypic properties of the cells. Relevant to this, we recently demonstrated, by mass cytometry and RNA sequencing, that gluten‐specific CD4^+^ T cells have a distinct phenotype in the blood and gut of CeD patients.^[^ ^19^ ^]^ Cells with the same phenotype are increased in the blood and/or inflamed tissue of patients with many different autoimmune conditions, including CeD, rheumatoid arthritis, systemic lupus erythematosus, type 1 diabetes, and systemic sclerosis.^[^ ^19^, ^20^, ^21^ ^]^ Renand and colleagues demonstrated that CD4^+^ T cells, phenotypically similar to gluten‐specific CD4^+^ T cells, are reactive to a soluble liver antigen in a subgroup of patients with autoimmune hepatitis. In rheumatoid arthritis, cells with a phenotype similar to that of gluten‐specific CD4^+^ T cells were termed peripheral T helper cells due to their expression of multiple markers associated with T‐cell help to B cells, ability to induce B cells into antibody‐producing plasma cells in vitro, and their lack of CXCR5,^[^ ^20^ ^]^ which distinguishes them from bona fide T follicular helper cells. We recently reported that in treated CeD at baseline before gluten challenge a fraction of the gluten‐specific CD4^+^ T cells express CXCR5 and that the cells upon in vivo activation express other markers, such as CD161, CD147, and CD132—that are not typically associated with T‐cell help.^[^ ^22^ ^]^ Thus, herein we have termed CD4^+^ T cells with this distinct phenotype T phenotypic (Tphe).

In this paper, we aimed to test in the setting of CeD, whether gluten‐specific CD4^+^ T cells may be isolated directly, solely based on this particular phenotype without the use of antigen‐stimulation or by use of HLA tetramers. To this end, we used an 11‐color flow cytometry panel that was developed based on our existing knowledge of the Tphe cell phenotype. The results, benchmarked by parallel isolation of HLA‐DQ:gluten peptide tetramer reactive (Ttet) cells, suggest that this approach indeed is feasible.

## 2 Results

### 2.1 Phenotypic markers that define Tphe cells by flow cytometry

**Figure 1:**
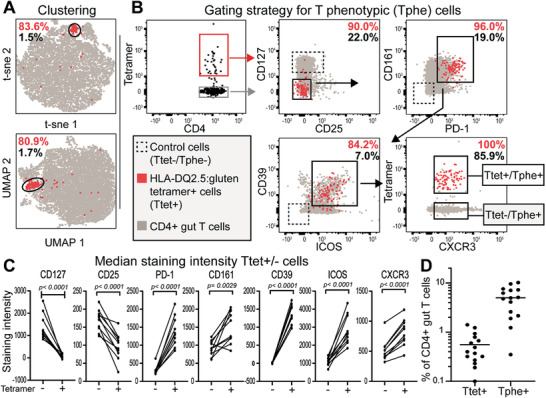
Characteristics and frequency of Tphe cells. a) t‐sne and UMAP plot of gut‐derived CD4^+^ T cells from a celiac disease (CeD) patient. Percentages indicate HLA‐DQ2.5:gluten‐tetramer^+^ (Ttet^+^) (red) and CD4^+^ gut (grey) T cells in general locating within the circled cluster. b) Gating strategy for CD4^+^ gut T cells to obtain the Tphe phenotype. c) Median staining intensity of the seven indicated cell‐surface markers defining the Tphe phenotype and used to produce the t‐sne and UMAP plots in (a). Only the samples from *n* = 10 untreated patients analyzed in the same Flow cytometer were used for this analysis. Paired *t*‐test was performed to obtain the *p*‐values. d) Frequency of Ttet^+^ and Tphe^+^ cells gated as in (b) in *n* = 15 untreated CeD patients.

From our previous study on the phenotypic features of gluten‐specific CD4^+^ gut T cells,^[^ ^19^ ^]^ we established an 11‐color flow panel (Table S1, Supporting Information). The flow panel includes lineage markers (CD3 and CD4) and seven phenotypic markers (CD127, CD25, PD‐1, CD161, ICOS, CD39, and CXCR3). In addition, it includes gluten‐HLA‐DQ2.5 tetramers to isolate positive control T (Ttet) cells. Here, we analyzed single‐cell suspensions prepared from biopsies of duodenal, inflammatory lesions of untreated CeD patients (Patient information in Table S2, Supporting Information). Analysis of the gut‐biopsy derived CD4^+^ T cells with UMAP and t‐sne, showed distinct clustering of >80% of the Ttet cells using the seven phenotypic markers, while excluding HLA‐DQ2.5:gluten‐tetramer staining (**Figure** **1**a). Remarkably, the distinct clustering of >80% is similar to our previous mass cytometry‐based t‐sne analysis of gluten‐specific CD4^+^ T cells involving 31 markers.^[^ ^19^ ^]^ The Ttet cells had a distinct expression of each of the chosen markers, as visualized in the gating strategy (Figure 1b) and by the median marker expression (Figure 1c). The T phenotypic^+^ (Tphe^+^) and Tphe^−^ cells were defined and sorted as CD127^−^/CD25^−^/PD‐1^+^/CD161^+^/ICOS^+^/CD39^+^/CXCR3^+^ cells and CD127^+^/PD1^−^/CD161^−^/ICOS^−^/CD39^−^ cells, respectively. On average, 0.6% of the total CD4^+^ gut T cells stained with HLA‐DQ2.5:gluten‐tetramers (representing five dominant gluten T‐cell epitopes in CeD), and 5.0% were Tphe^+^ cells (Figure 1d). Similarly, we have previously demonstrated by mass cytometry that the frequency of Tphe^+^ cells among CD4^+^ gut T cells was 5% in untreated CeD patients, in contrast to 0.1% in healthy controls.^[^ ^19^ ^]^ Taken together, the seven phenotypic markers clearly separate gluten‐HLA‐DQ2.5‐tetramer binding cells from most other CD4^+^ T cells in the inflamed gut tissue of CeD patients.

### 2.2 Ttet^−^/Tphe^+^ T cell population contains gluten‐reactive T cells

**Figure 2:**
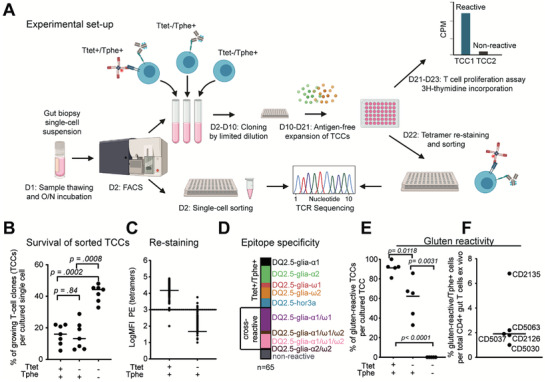
T cell proliferation assay and experiment layout. a) Cryopreserved single‐cell suspension from celiac disease (CeD) gut biopsies were thawed, incubated overnight, and stained on the day 2 with a cocktail of five HLA‐DQ2.5:gluten tetramers and a mix of antibodies for FACS sorting. Three populations (Ttet^+^/Tphe^+^, Ttet^−^/Tphe^+^, Ttet^−^/Tphe^−^) were sorted either for T‐cell receptor sequencing (single‐cell) and/or for generating T cell clones (TCCs). The TCCs were generated by performing cloning by limited dilution followed by antigen‐free expansion. The TCCs were then screened for antigen‐specific proliferative responses in a T cell proliferation assay using 3H‐thymidine incorporation, recorded as counts per minute (cpm), and expressed as stimulation index (SI) (cpm after gluten stimulation/cpm after solvent stimulation). TCCs with SI > 1.8 were considered reactive. TCCs were also re‐stained with the cocktail of HLA‐DQ2.5:gluten tetramers used during sorting. TCCs were then sorted in 96‐well plates for TCR sequencing. b) Frequency of successfully generated TCCs per cultured single CD4^+^ gut T cell from each of the indicated FACS‐sorted populations Ttet^+^/Tphe^+^, Ttet^−^/Tphe^+^, Ttet^−^/Tphe^−^ (*n* = 7 untreated CeD patients). c) HLA‐DQ2.5:gluten‐tetramer re‐staining intensity (log median fluorescence intensity – MFI) summarized for TCCs generated from Ttet^+^/Tphe^+^ sorted (*n* = 83 TCCs) and Ttet^−^/Tphe^+^ sorted (*n* = 209 TCCs) CD4^+^ T cells from five untreated CeD patients. The horizontal bar indicates the median value. d) Fine specificity of Ttet^+^/Tphe^+^ sorted TCCs (*n* = 65 TCCs) tested with peptides representing five immunodominant epitopes that were used for HLA‐DQ2.5:gluten‐tetramer sorting (SI > 1.8) e) Frequency of TCCs with proliferative response against deamidated gluten antigen per total number of cultured TCCs from indicated populations in vitro. Each dot represents the percentage within a separate participant. f) Frequency CD4^+^ gut T cells with proliferative response against deamidated gluten in vitro (*n* = 5 untreated CeD patients). The horizontal bar indicates the median value. Horizontal bars indicate the median value. *p*‐Values calculated by two‐tailed paired *t*‐tests in (b) and (e).

The average Ttet^+^ to Tphe^+^ ratio was 3:20, calculated from the frequency of Ttet^+^ and Tphe^+^ cells (Figure 1d). Thus, we aimed to determine the extent of gluten reactivity within Tphe^+^ cells that did not stain with the HLA‐DQ2.5:gluten tetramers. We performed FACS sorting (Figure 1) and isolated three populations of T cells (Ttet^+^/Tphe^+^, Ttet^−^/Tphe^+^, Ttet^−^/Tphe^−^) from the gut biopsies of untreated CeD patient (**Figure** **2**a). This sorting resulted in three different sets of T‐cell clones (TCCs) from each patient, generated by limited dilution and antigen‐free expansion. When assessing the cloning efficiency of the three populations, we found that significantly more cells from the control population (Ttet^−^/Tphe^−^ cells) compared to the two Tphe^+^ cell populations survived the cloning process (Figure 2b). There was, however, no significant difference between Ttet^+^/Tphe^+^ and Ttet^+^/Tphe^−^ populations in this regard (Figure 2b). When verifying the HLA‐DQ2.5:gluten‐tetramer binding of each generated TCC, almost all the Ttet^+^/Tphe^+^ sorted cells (81/83) but very few Ttet^−^/Tphe^+^ sorted cells (6/209) were re‐stained with the cocktail of the HLA‐DQ2.5:gluten tetramers used for sorting (Figure 2c, staining panel in Table S3, Supporting Information). The specificity of the HLA‐DQ2.5:gluten‐tetramer sorting was further confirmed by the observation that 90.8% of the Ttet^+^/Tphe^+^ sorted TCCs (*n* = 65) gave a proliferative response in vitro to at least one of the five immunodominant epitopes represented by the HLA‐DQ2.5:gluten‐tetramers (Figure 2d). We also observed some cross‐reactivity within these five epitopes, as previously described.^[^ ^23^, ^24^ ^]^ However, to find whether gluten‐reactive cells also could be isolated directly without HLA‐DQ2.5:gluten‐tetramers, we analyzed the fraction of gluten reactivity within the Ttet^−^/Tphe^+^ TCCs. Indeed, a median of 65.3% of the Ttet^−^/Tphe^+^ TCCs was reactive to gluten, compared to 92.3% within the TCCs generated from Ttet^+^/Tphe^+^ cells and 0% within the TCCs generated from Ttet^−^/Tphe^−^ cells, as assessed by T‐cell proliferation to deamidated gluten (Figure 2E). Significantly more TCCs generated from Ttet^+^/Tphe^+^ cells versus Ttet^−^/Tphe^+^ cells were gluten reactive. Based on the Tphe‐cell frequency among CD4^+^ T cells (Figure 1d) and the fraction of gluten reactivity within the generated TCCs (Figure 2e) we calculated the percentage of gluten‐specific cells per total CD4^+^ gut T cells to be 1.9% in the five untreated CeD patients from whom TCCs were generated (Figure 2f). Taken together, gluten‐specific CD4^+^ T cells can be sorted directly from the gut of CeD patients at high frequencies only based on a limited number of phenotypic markers and without the use of HLA tetramers.

### 2.3 Ttet^−^/Tphe^+^ cells demonstrate clonal expansion and sharing of TCR sequences across patients

We performed high‐throughput sequencing of rearranged TCR‐*α* and TCR‐*β* genes of Ttet^+^/Tphe^+^, Ttet^−^/Tphe^+^, and Ttet^−^/Tphe^−^ CD4^+^ cells derived either from single cells sorted ex vivo or from TCCs generated in vitro (*n* = 12 untreated CeD patients). Only cells with productive paired TCR‐*αβ* chains were included for downstream analysis resulting in 1535 cells of which 1175 were generated from single cells and 360 were generated from TCCs. The number of cells and clonotypes (identical nucleotide TCR‐*αβ* sequences) isolated from each donor is shown in **Table** **1**. We used these results to interrogate whether TCR sequence data may assist in identifying T cells that are candidate pathogenic cells. Specifically, within each subset we looked for a) *TRAV* and *TRBV* usage, b) signs of clonal expansion, and c) the presence of TCRs that are shared across patients (i.e., public sequences). We interrogated whether these sequences are prototypic of gluten‐specific CD4^+^ T cells doing a comparison with an established collection of public TCRs used by gluten‐specific T cells.^[^ ^25^, ^26^ ^]^

**Table 1:** Information on the number of cells and clonotypes from each donor used for TCR analysis

Patients	Status	Sample/TCR sequencing	Ttet+/Tphe+		Ttet+/Tphe‐		Ttet^‐^/Tphe‐	
			Cells	Clonotypes	Cells	Clonotypes	Cells	Clonotypes
CD2126		Direct sorting of T cells from gut biopsies for single cell TCR sequencing (single cell)	0	0	21	21^b)^	12	12
CD5037			20	19^f)^	43	42^d)^	0	0
CD5065			68	44	154	125	83	78
CD5058			10	10	60	57	35	35
CD5060			0	0	38	37	80	75
CD5028			19	19	46	44	74	72
CD2326	UCeD^a)^		63	48	148	143	73	73
CD2219			11	10	117	114	0	0
		
CD2126		Sorting T cells of T cell clones generated from gut biopsies for TCR sequencing (TCC)	21	18	49	41^c)^	10	10
CD2135			14	12	46	43	10	10
CD5030			10	10	6	6	33	30
CD5037			13	10^g)^	66	61^e)^	21	21
CD5063			20	20	0	0	0	0
CD5038			1	1	8	8	32	32
TOTAL *single‐cell sorted* (1175 cells, 1078 clonotypes)			191	150	627	583	357	345
TOTAL *TCC* (360 cells, 333 clonotypes)			79	71	175	159	106	103
TOTAL (1535 cells, 1403^h)^ clonotypes)			270	220	802	736	463	448

^a)^ Untreated celiac disease (UCeD)

^b)^ 2 clonotypes comprising of 2 cells were also found among cells sequenced as single cells

^c)^ 2 clonotypes comprising of 3 cells were also found within T cell clones (TCCs)

^d)^ 4 clonotypes comprising of 5 cells were also found within single cells

^e)^ 4 clonotypes comprising of 5 cells were also found within TCCs

^f)^ 1 clonotype comprising of 1 cell was also found within single cells

^g)^ 1 clonotype comprising of 1 cell was also found within TCCs

^h)^ 1 clonotype comprising of 2 cells was also found in CD5060 Ttet+/Tphe+ and CD5060 Ttet‐/Tphe + from single‐cell sorted cells.

**Figure 3:**
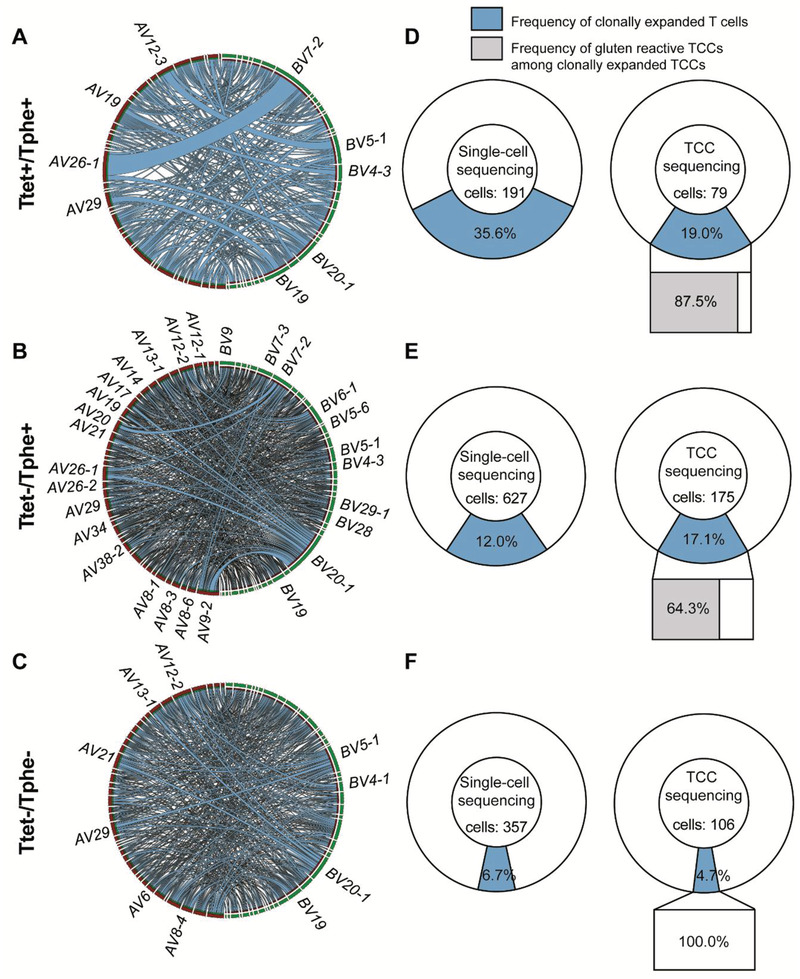
Clonal expansion and V‐gene usage of T cells based on their phenotype. a,d) Analysis of Ttet^+^/Tphe^+^ cells, b,e) Ttet^−^/Tphe^+^ cells and c,f) Ttet^−^/Tphe^−^ cells. a–c) Circos plots representing chain pairing of *TRAV* (red) and *TRBV* (green) in clonotypes of single‐cell derived and T‐cell clones (TCCs)‐derived CD4^+^ T cells in each subset. *TRAV* and *TRBV* gene labels are displayed only for the genes that are involved in gene pairs of at least four different clonotypes. Thus, many used gene pairs are not labelled to avoid overloaded figures. d–f) Donut charts illustrate the clonal expansion among single‐cell derived and TCC‐derived T cells in each subset. In the doughnut charts, the blue sections represent the frequency of clonally expanded T cells. In bar charts below the rightmost doughnut plots, the gray and white bars represent the frequency of gluten‐reactive and non‐gluten‐reactive TCCs, respectively, among the clonally expanded TCCs.

The usage of *TRAV* and *TRBV* genes for each cell subset for all 12 donors collectively is illustrated by Circos plots (**Figure** **3**a–c). In the Ttet^+^/Tphe^+^ subset, a biased usage of *TRAV26‐1:TRBV7‐2* and *TRAV12‐3:TRBV5‐1* was particularly apparent (Figure 3a). Of note, the *TRAV26‐1:TRBV7‐2* bias is a signature for T cells specific for the DQ2.5‐glia‐*α*2 epitope.^[^ ^13^, ^23^, ^25^, ^27^ ^]^ For the Ttet^−^/Tphe^+^ cells some of the same biases as seen for the Ttet^+^/Tphe^+^ cells were present, while for Ttet^−^/Tphe^−^ cells no clear biases could be observed (Figure 3b,c).

We have previously observed the presence of clonally expanded cells among HLA‐DQ2.5:gluten tetramer‐sorted cells.^[^ ^12^, ^23^, ^24^ ^]^ We thus looked for clonal expansion within each subset defining clonal expansion as the presence of two or more cells with identical TCR‐*αβ* the sequence within each donor. We found that Ttet^+^/Tphe^+^ cells displayed the highest frequency of clonally expanded cells and here several clones were represented by more than two cells (Figure 3d). Importantly, we found clonally expanded cells also in the Ttet^−^/Tphe^+^ although to a lesser extent than for Ttet^+^/Tphe^+^ cells (Figure 3e). The Ttet^−^/Tphe^−^ cells had the lowest frequency of clonally expanded cells (Figure 3f). Among the expanded Ttet^+^/Tphe^+^ and Ttet^−^/Tphe^+^ clonotypes, 87.5% and 64.3%, respectively, were reactive to deamidated gluten in the T‐cell proliferation assays (Figure 3d,e). Despite some few clonally expanded Ttet^−^/Tphe^−^ TCCs, none of these were reactive to deamidated gluten antigen (Figure 3f). We then looked at *TRAV:TRBV* pairs used by clonally expanded cells. Of the 270 Ttet^+^/Tphe^+^ cells, we found 34 expanded clonotypes of which seven clonotypes carried either TCR‐*α*, TCR‐*β* or TCR‐*αβ* sequences that were present within the collection of public TCRs (Figure S1A, Supporting Information). Among 802 Ttet^−^/Tphe^+^ cells, we found 48 expanded clonotypes of which one clonotype carried a TCR‐*β* sequence matching a sequence in the public TCR collection (Figure S1B, Supporting Information). Among 463 Ttet^−^/Tphe^−^ single‐sorted T cells, none of 14 expanded clonotypes matched any sequences within the public TCR collection (Figure S1C, Supporting Information). The sequences of clonotypes using TCRs that matched public, gluten‐specific TCR sequences are shown in Table S4 (Supporting Information). The frequency of expanded clonotypes was twofold higher in Ttet^+^/Tphe^+^ versus Ttet^−^/Tphe^+^ cells (34/270 vs 48/802), yet in the former subset, the frequency of TCRs matching sequences in the public TCR collection was much higher (16/270 vs 2/802). This likely relates to the selection of TCRs imposed by the HLA‐DQ2.5:gluten‐tetramers.

We also analyzed for the presence of shared TCRs across patients scoring for TCR‐*α*, TCR‐*β*, or paired TCR‐*αβ* sequences that were identical at the amino acid level in at least two of the 12 patients. In the Ttet^+^/Tphe^+^ subset, we observed five shared sequences, specifically two TCR‐*α* and three TCR‐*β* sequences (**Table** **2**). All these five shared sequences were found in the collection of public, gluten‐specific TCR sequences.^[^ ^25^, ^26^ ^]^ Thus, these sequences most likely represent prototypical gluten‐specific CD4^+^ T cells. Three of these sequences were present among the generated TCCs; two that we found to respond to deamidated gluten antigen and one that was unresponsive. A response to gluten in all TCCs could here be expected. In the Ttet^−^/Tphe^+^ populations, we identified 12 shared TCR sequences, specifically eight TCR‐*α* and four TCR‐*β* sequences (Table 2). Two of these 12 shared sequences were found in the collection of public, gluten‐specific TCR sequences. Six of the 12 shared TCR sequences were represented among the generated TCCs. Four of these TCCs were reactive to gluten stimulation, while two were not. No shared sequences were observed within the Ttet^−^/Tphe^−^ subset.

**Table 2:** TCR amino acid sequences identified to be shared across 12 CeD patients within the Ttet^+^/Tphe^+^ and Ttet^−^/Tphe^+^ subsets

Phenotype	Shared TCR*α*/TCR*β* sequences	Presence and reactivity^a)^												^b)^Previously reported dataset of public gluten‐specific TCRs;^c)^ number of CeD patients
		CD2126	CD2135	CD2219	CD2326	CD5028	CD5030	CD5037	CD5038	CD5058	CD5060	CD5063	CD5065	
Ttet^+^/Tphe^+^	AV4_LVGGSGGYNKLI_AJ4						R2							O, ^[^ ^25^ ^]^; 13
	AV41_AVEGGSNYKLT_AJ53													O, ^[^ ^25^ ^]^; 10
	BV7‐2_ASSIRATDTQY_BJ2‐3											R1		O, ^[^ ^25^ ^]^; 9
	BV7‐3_ASSIRSTDTQY_BJ2‐3							NR1						O, ^[^ ^25^ ^]^; 14
	BV20‐1_SASRTSGRAGDEQF_BJ2‐1													O, ^[^ ^26^ ^]^; 6
Ttet^−^/Tphe^+^	AV9‐2_ALSDQGSSASKII_AJ3*													NO
	AV9‐2_ALSDPTGTASKLT_AJ44													O, ^[^ ^25^ ^]^; 4
	AV10_VVSGGYYGGSQGNLI_AJ42													NO
	AV12‐1_VVNPGGGNKLT_AJ10		NR1											NO
	AV20_AVPNAGGTSYGKLT_AJ52	NR1												NO
	AV22_AVEREGAQKLV_AJ54	R3												NO
	AV36_AVGQGAQKLV_AJ54													NO
	AV38‐2_AYRSEQGAQKLV_AJ54													NO
	BV7‐3_ASSQGQDTEAF_BJ1‐1							R1						O, ^[^ ^25^ ^]^; 5
	BV7‐3_ASSLTVTDTQY_BJ2‐3		R1											NO
	BV20‐1_SASDSLNTEAF_BJ1‐1*													NO
	BV20‐1_SASRQVADTQY_BJ2‐3							R1						NO

^a)^ The grey filling represents patients that express the indicated public TCR sequences. Some sequences were expressed by T cell clones (TCCs) that were tested for gluten reactivity. For these sequences, the gluten reactivity is indicated as NR; not reactive or R; reactive followed by the number of TCCs

^b)^ The rightmost column denotes whether the public sequence have been O; observed or NO; not observed in a collection of previously defined public sequences of gluten‐specific CD4^+^ T cells

^c)^ TCR‐*α*, TCR‐*β*, and paired TCR‐*αβ* sequences using identical V‐/J‐genes and identical CDR3 amino acid sequences found in the same cell subset of at least two patients were defined as public.

## 3 Discussion

Despite well‐established HLA class II associations in several autoimmune conditions,^[^ ^28^ ^]^ the disease‐driving antigen recognized by CD4^+^ T cells for most diseases remains elusive. Lack of knowledge about the T‐cell driving antigens makes available techniques for isolation of disease‐specific T cells, such as the MHC/HLA tetramer technology, not relevant for the study of such diseases. In this study, we demonstrate that CD4^+^ T cells specific to the disease‐driving antigen in CeD can be isolated directly and in high numbers from the inflammatory lesion, by using FACS and a small set of phenotypic markers without the use of HLA tetramers. T cells with the investigated phenotype (i.e., Tphe^+^ cells), have increased frequency in the blood of several autoimmune conditions including rheumatoid arthritis and such T cells have been found to display autoreactivity in autoimmune hepatitis.^[^ ^20^, ^29^, ^30^ ^]^ Thus, our findings represent a proof‐of‐concept for phenotype‐based isolation of disease‐specific CD4^+^ T cells in CeD that may be helpful in the identification of disease‐relevant CD4^+^ T cells in other autoimmune conditions.

Our study included staining with HLA‐DQ2.5:gluten‐tetramers for benchmarking. We generated TCCs from Tphe cells being either Ttet^+^/Tphe^+^ or Ttet^−^/Tphe^+^ cells. From both populations gluten‐reactive TCCs were generated, yet, as could be expected, significantly more gluten‐reactive TCCs were generated from Ttet^+^/Tphe^+^ cells versus Ttet^−^/Tphe^+^ cells. The results indicate that sorting by phenotype alone (i.e., Tphe cells) would give the majority of gluten‐reactive TCCs. Transferred to a disease with no knowledge of disease‐driving antigen, sorting of Tphe cells should give TCCs of which many hopefully have specificities for the disease‐driving antigen. It is striking that 65.3% of the Ttet^−^/Tphe^+^ cells were reactive to gluten as in this sorted cell population T cells specific to the most dominant T‐cell epitopes in CeD were excluded by staining with HLA‐DQ2.5:gluten‐tetramers. Possibly, TCCs scored negative for gluten reactivity still could be reactive to gluten if their epitopes would not be present in the gluten antigen used. Gluten is a complex mixture of hundreds of individual proteins, and there is not necessarily a complete representation of all gluten proteins in our antigen preparation. Thus, 65.3% is an underestimate for the number of gluten‐specific cells within gut‐derived CD4^+^ Tphe^+^ cells in CeD patients. Whether there also exist Ttet^−^/Tphe^+^ cells being specific for a non‐gluten antigen that is relevant to the pathogenesis of CeD remains speculation at this point.

Single‐cell TCR sequencing could be useful to identify T cells of particularly interesting specificities, for instance in a quest for identifying unknown disease‐driving antigens. Observing clonally expanded cells could be a lead. In our study, the frequency of expanded clonotypes was twofold higher in Ttet^+^/Tphe^+^ versus Ttet^−^/Tphe^+^ cells. In addition, the frequency of gluten‐signature TCRs used by T cells responsive to gluten epitopes of the HLA‐DQ2.5:gluten‐tetramers is higher among Ttet^+^/Tphe^+^ cells (16/270 vs 2/802). This is not surprising as these cells were sorted using HLA‐DQ2.5:gluten‐tetramers representing five immunodominant gluten epitopes. Clonally expanded T cells within the Ttet‐/Tphe+ subset are particularly interesting as they likely represent T cells of importance in CeD pathogenesis. The presence of shared TCR sequences across patients could be another lead. This notion is supported by our observation that within the Ttet^+^/Tphe^+^ subset the shared TCRs all had sequences that matched sequences in the collection of public, gluten‐specific TCRs.^[^ ^25^ ^]^ Importantly, we also observed the existence of shared TCRs in the Ttet^−^/Tphe^+^ subset, yet with less matches in the collection of public, gluten‐specific TCRs.^[^ ^25^, ^26^ ^]^ The four shared TCRs of gluten‐reactive TCCs that did not match previously identified public TCRs (Table 1), would be candidates for discovering new important gluten T‐cell epitopes.

In our previous mass cytometry‐based study and the current flow cytometry‐based study, Tphe cells made up 5% of the total CD4^+^ gut T cells in the celiac lesion, as defined by manual gating. Using t‐sne and UMAP analysis, Tphe cells were determined to make up 1.5% and 1.7% of CD4^+^ T cells in these two studies, respectively. Relevant to this, we estimated in the five CeD patients from whom TCCs have established the frequency of gluten‐specific cells among CD4^+^ T cells to be 1.9%. The estimated frequency is in line with previous reports using combinations of HLA‐DQ2.5:gluten‐tetramers and direct cloning from gut‐derived CD4^+^ T cells.^[^ ^31^, ^32^ ^]^ We observed that significantly less cells of the Ttet^+^/Tphe^+^ and Ttet^−^/Tphe^+^ populations survived the in vitro cloning procedure (Figure 2b). Gluten‐specific gut T cells do not express typical markers associated with so‐called “exhausted T cells,” and they appear to play an active role in the celiac lesion through the production of cytokines such as IFN‐*γ*, IL‐21, and CXCL13.^[^ ^19^, ^33^ ^]^ Nonetheless, the lower capacity to survive in vitro expansion suggests that the percentage of cells specific to a test antigen will be underestimated when measuring the frequency of antigen responsive TCCs in a recall assay.

Using the knowledge of disease‐driving CD4^+^ T cells in CeD as a test case, we demonstrate that it is possible to isolate disease‐driving pathogenic T cells solely based on phenotypic markers. We also provide evidence that single‐cell TCR sequencing with detection of clonally expanded T cells and the presence of shared TCRs sequences across patients may provide additional clues for identifying T cells of particular interest. The findings bear promise for isolation and characterization of disease‐driving pathogenic CD4^+^ T cells in conditions where cells with such phenotype are increased.

## 4 Experimental Section

Detailed Experimental Section for the current study is provided in the Supporting Information. The TCR sequencing raw data generated in this study are uploaded to the European Genome‐phenome Archive (EGAS00001005582).^[^ ^34^ ^]^ Other data of this study are available from the authors upon reasonable request.

The study was approved by Regional Committee for Medical and Health Research Ethics South‐East Norway (project #6544). All participants gave informed written consent.

## Conflict of Interest

The authors declare no conflict of interest.

## Author Contributions

A. C., S. D.‐K., and M. C. contributed equally to this work. A. C. was associated with conceptualization, funding acquisition, investigation, methodology, data curation, formal analysis, supervision, visualization, writing original draft, review, and editing. S. D.‐K. was associated with investigation, methodology, data curation, formal analysis, supervision, visualization, writing original draft, review, and editing. M. C. was associated with investigation, methodology, data curation, formal analysis, visualization, writing original draft, review, and editing. J. J. was associated with resources, data curation, review, and editing. K. E. A. L. was associated with resources, data curation, review, and editing. L. M. S. was associated with funding acquisition, data analysis, supervision, review, and editing. The order of the three first authors was based on the conceptualization, planning of the project, and the writing of the manuscript.

## Supporting information

Supporting Information

## Data Availability

The data that support the findings of this study are openly available in European Genome‐phenome Archive at https://ega‐archive.org/studies/EGAS00001005582, reference number 34.
