# Supplementary material for: Phenotype‐Based Isolation of Antigen‐Specific CD4+ T Cells in Autoimmunity: A Study of Celiac Disease
Source: Adv Sci (Weinh). 2022 Feb 4;9(10):2104766. doi: 10.1002/advs.202104766 (PMC8981484; doi:10.1002/advs.202104766)
Supplement: Supplementary file 1 — Supporting Information [file ADVS-9-2104766-s001.pdf]

ADVANCED SCIENCE
Open Access

Supporting Information

for *Adv. Sci.*, DOI 10.1002/advs.202104766

Phenotype-Based Isolation of Antigen-Specific CD4$^+$ T Cells in Autoimmunity: A Study of Celiac Disease

Asbjørn Christophersen, Shiva Dahal-Koirala, Markéta Chlubnová, Jørgen Jahnsen, Knut E. A. Lundin and Ludvig M. Sollid**

ADVANCED SCIENCE
Open Access

Supporting Information

for *Adv. Sci.*, DOI: 10.1002/advs.202104766

Phenotype-based isolation of antigen-specific CD4+ T cells in autoimmunity: A study of celiac disease

Asbjørn Christophersen, Shiva Dahal-Koirala, Markéta Chlubnová, Jørgen Jahnsen, Knut E. A. Lundin and Ludvig M. Sollid

Title: Phenotype-based isolation of antigen-specific CD4+ T cells in autoimmunity: A study of celiac disease

Authors: *Asbjørn Christophersen*[*], *Shiva Dahal-Koirala, Markéta Chlubnová, Jørgen Jahnsen, Knut E. A. Lundin and Ludvig M. Sollid*[*]

Dr. Asbjørn Christophersen, Dr. Shiva Dahal-Koirala and Markéta Chlubnová contributed equally.

*E-mail: asbjoc@medisin.uio.no; l.m.sollid@medisin.uio.no

Supporting information

Supplementary Materials and Methods

Supplementary Figure

- Figure S1. Stack bar representation of clonal distributions

Supplementary Tables

- Table S1. Antibody panel for ex-vivo cell sorting.
- Table S2. Patient information.
- Table S3. Antibody panel for re-staining of TCCs (used in Figure 2C).
- Table S4. Use of public TCRs[a)] by clonally expanded T cells identified within the Ttet+/Tphe+ and Ttet-/Tphe+ subset of T cells.

Supplementary Materials and methods

Patient information

All samples were from patients giving informed written consents and visiting the Endoscopy Unit at Oslo University Hospital - Rikshospitalet or the Endoscopy Unit at Akershus University Hospital, for the endoscopic routine procedure for diagnosis of celiac disease (CeD). Detailed information about patients and samples used in this study can be found in **Table S2**. The patients fulfilled the diagnostic criteria for CeD,[1] having increased levels of

antibodies to deamidated gluten peptide (DGP) and transglutaminase 2 (TG2) and small intestinal biopsies with a Marsh score 3. All patients were selected to express HLA-DQ2.5 (that is, *DQA1*05:01* and *DQB1*02:01).*

Generation of single-cell suspension

The gut biopsies were processed and cryopreserved as described previously.[2] Briefly, duodenal biopsies (8-12 biopsies from each patient) were sampled into ice-cold media RPMI 1640 (Sigma). Biopsies were incubated 2 x 10 min at 37°C with continuous rotation in 2mM EDTA (Sigma)/2% FCS/PBS (Sigma) solution to remove epithelial cells. Samples were further digested with collagenase buffer (1mg/ml) collagenase, type H, (Sigma)/2%FCS/PBS (Sigma) for 45min at 37°C with continuous rotation. In the last step, samples were homogenized with syringe (BD Microlance - 18G, BD Biosciences) before filtering with 40µm cell strainer (Falcon) and subsequent wash. The single-cell suspension was then cryopreserved.

HLA-DQ2.5:gluten-tetramers

We used in-house produced gluten-HLA:DQ2.5 molecules,[3] representing immunodominant T-cell epitopes derived from gluten,[4] i.e. DQ2.5-glia-α1a (QLQPFPQPELPY), DQ2.5-glia-α2 (PQPELPYPQPE, underlined 9-mer core amino acid sequence), DQ2.5-glia-ω1 (QQPFPQPEQPFP), DQ2.5-glia-ω2 (FPQPEQPFPWQP) and DQ2.5-hor3a (PIPEQPQPYPQ).[5] These peptides were covalently linked with HLA-DQ2.5 molecules, which were later biotinylated followed by conjugation with phycoerythrin (PE)-labelled streptavidin (Invitrogen), as previously described.[3, 6] A cocktail of PE-conjugated gluten-HLA:DQ2.5 molecules representing the five gluten epitopes was used for cell staining. We also prepared a negative control HLA-DQ2.5-tetramer that contained the invariant chain peptide CLIP2 (MATPLLMQALPMGAL) (DQ2-CLIP2) peptide. This HLA-DQ2.5-tetramer was conjugated with allophycocyanin (APC) (ProZyme).

Staining

Single-cell suspension was thawed and re-suspended in culture medium (10% human serum (HS), RPMI 1640, (Sigma), supplemented with penicillin-streptomycin (Sigma) and 0.01M β-Mercapthoethanol (Sigma)) and incubated overnight in a humidified incubator at 37°C with 5% CO_2. Overnight rested single-cell suspension was incubated for 15 min with Benzonase Nuclease (Sigma) (1:10 000) in flow buffer (2mM EDTA (Sigma) PBS (Sigma)/PBS 1:1 and 2%FCS) at room temperature and then filtered through the 40 µm cell strainer (Falcon) and washed. To prevent unspecific binding, samples were incubated with FcR blocking reagent (Miltenyi) (200ul of cell suspension in flow buffer) for 15min on ice. Washed samples were then incubated with a cocktail of PE-conjugated HLA-DQ2.5:gluten tetramers representing DQ2.5-glia-α1a, DQ2.5-glia-α2, DQ2.5-glia-ω1, DQ2.5-glia-ω2 and DQ2.5-hor3a (10 µg/ml of each) for 40 min with continuous rotation at room temperature. Lastly, washed samples were stained with the antibody panel (**Table S1**) for 20 min on ice in dark (staining volume 50µl). Samples were then washed and re-suspended in flow buffer prior to sorting.

Cell sorting from single-cell suspension

CD4+ T cells of interest were sorted using a FACs Aria III cell sorter (BD Biosciences) and analyzed using FlowJo software (Three Star). Single CD3+ CD4+ T cell lymphocytes that were dump negative (CD11c-, CD14-, CD19-, CD56-) were further sorted into 3 subpopulations, depending on whether they stained with HLA-DQ2.5:gluten tetramers and whether they held the distinct T-cell phenotype, ICOS+, CXCR3+, CD39+, CD25-, PD-1+, CD127-, CD161+, here termed Tphe. The cells were sorted into tubes in order to generate TCCs or in Hard-Shell 96-well plates (BioRad) for single-cell TCR sequencing.

Generation of T-cell clones

TCCs were generated as previously described.[7] Briefly, T cells with desired phenotype were sorted in a tube with standard feeder mix (peripheral mononuclear cells from 3 healthy donors, irradiated at 60Gy). TCCs were generated by limiting dilution (1 TCC/3 wells in volume of 20µl) using Terasaki plates (VWR). Antigen-free culture medium, consisting of 10% HS, RPMI 1640 (Sigma), supplemented with penicillin-streptomycin (Sigma) and 0.01M β-mercapthoethanol (Sigma), together with irradiated peripheral mononuclear cells ($1x10^6$/ml,

feeder mix) and IL-2 (10 IU/ml) (Amersham Pharmacia Biotech AB), IL-15 (1 ng/ml) (R+D Systems), PHA (1 µg/ml) (Murex), was used for generating TCCs. The TCCs were cultured for 10 days in Terasaki plates placed in a moist chamber inside a humidified incubator at 37°C with 5% CO_2. The growing TCCs were then subjected to further expansion using in culture medium for 10 days before testing for reactivity with gluten and re-staining with HLA-DQ2.5:gluten tetramers and the negative control HLA-DQ2.5-tetramer.

Antigens

We used previously described protocol for proteolytic digestion of gluten.[7] Cereal antigens of gluten were prepared in-house from wheat (Møllerens - siktet hvetemel). The isolated antigens were digested with chymotrypsin (Sigma) and deamidated with in-house prepared TG2 (50µg/ml) before they were added to the T cell proliferation assay. For mapping of epitope specificities, we used the DQ2.5-glia-α1a-epitope peptide (QLQPFPQPELPY, underlined 9mer core sequence), the DQ2.5-glia-α2-epitope (PQPELPYPQPQL) (both from Research Genetics), the DQ2.5-glia-ω1 epitope (PQQPFPQPEQPFP), the DQ2.5-glia-ω2 epitope (FPQPEQPFPWQP) and the DQ2.5-hor3a epitope (PEQPIPEQPQPYPQQP) (all three from GenScript).

T-cell proliferation assay

For the antigen-dependent proliferation assays, we used a well-established protocol.[7] T-cell proliferation assay was performed in clear 96-well round bottom plates (Corning). On day 1 of the assay, 75ul of irradiated (75Gy) B-cells (Raji cells, HLA-DQ2.5+) were added to each well together with 25ul of in TRIS-HCl (solvent) diluted antigen (10µM) (gluten)) or negative control (just the solvent without antigens), where each antigen/negative control was tested in triplicate with each TCC. On day 2, 50ul (approximate concentration $1x10^6$/ml) of TCCs was added to plates. On day 3, 20ul of ^{3}H-thymidine (Hartman Analytics) diluted in 0.9% NaCl solution (Braun) (1 µCi/well (0.037 MBq/well) was added to each well and after 24h, the stimulation with antigen was measured by the uptake of ^{3}H-thymidine. Cells were harvested using automated harvester (Mach III; TomTec) and the radioactivity of thymidine, incorporated during cell division, was measured by liquid stimulation counting (Wallac

MicroBeta TriLux 1450; PerkinElmer) as counts per minute (CMP). Thereafter, we calculated the stimulation index (SI) using the following formula:

mean of proliferation count (CPM_A) for a given antigen / mean of proliferation count (CPM_{NC}) for the negative control (solvent) = SI

Re-staining of TCCs and sorting of TCCs for TCR sequencing

Washed TCCs were incubated with a cocktail of PE-conjugated HLA-DQ2.5:gluten tetramers representing DQ2.5-glia-α1a, DQ2.5-glia-α2, DQ2.5-glia-ω1, DQ2.5-glia-ω2 and DQ2.5-hor3a (10 µg/ml of each) and negative control HLA-DQ2.5-tetramers APC conjugated DQ2.5-CLIP2 (10 µg/ml) for 40 min at room temperature (RT). Samples were then washed with ice-cold flow buffer and stained with the antibody panel (**Table S3**) for 20 min on ice in dark (staining volume 30µl). Lastly, washed samples were resuspended in flow buffer prior to sorting. TCCs were sorted in Hard-Shell 96-well plates (BioRad) using a FACs Aria II cell sorter (BD Biosciences) for TCR sequencing. The re-stained TCCs were analyzed on LSR Fortessa (BD Biosciences). Acquired data were analyses using FlowJo software (Three Star).

TCR sequencing

A previously established protocol for single-cell paired TCR-αβ sequencing using multiplex primers of TRAV and TRBV genes and nested PCR amplification was used for TCR sequencing.[2] A minor deviation from the originally described protocol was that 3 ul of cDNA was used instead of 1 ul. For sequencing of TCRs from the TCCs, 6-15 cells were sorted in each well. The amplicon library was sequenced using Illumina MiSeq (250 bp PE) platform at the Norwegian Sequencing Centre (Oslo University Hospital).

Data processing

The TCR sequencing data was processed and analyzed as described in.[8] Briefly, MiXCR was used to assemble the raw Illumina sequencing reads into rearranged TCR sequences, which were then submitted to the IMGT High V-QUEST.[9, 10] The IMGT output files together with the metadata associated with each sequence were uploaded to our in-house Immune Receptor

Information System (IRIS), a data analysis software as well as a data repository.[8] In IRIS, the sequences were filtered to remove unproductive sequences as well as sequences with less than 50 read counts. Further, only valid cells, i.e. cells that comprises one or two TCRα and TCRβ sequences, maximum three sequences, were included to prevent the inclusion of accidental doublets. Once these filters were implemented, IRIS was used to analyze any specific dataset (based on the metadata) either for browsing the sequences or as an input for analyzing the V-gene usage, clonal expansion and sharing of sequences. The TCR sequencing raw data generated in this study are uploaded to the European Genome-phenome Archive, https://www.ebi.ac.uk/ega under the accession number EGAS00001005582. Flow cytometry data were analyzed using FlowJo software (Three Star). All the figures were generated in Adobe Illustrator.

Reference public TCRs used by gluten-specific CD4+ T cells

We have previously identified 325 public TCR sequences (i.e. TCRα: 145, TCRβ: 102 and paired TCRαβ: 78) of T cells specific to four immunodominant gluten epitopes, i.e. DQ2.5-glia-α1a, DQ2.5-glia-α2, DQ2.5-glia-ω1 and DQ2.5-glia-ω2.[8] Similarly, we have identified 12 (public TCR sequences i.e. TCRα:3, TCRβ:6 and paired TCRαβ:3) of T cells specific to the immunodominant gluten epitope, DQ2.5-glia-hor3.[11] Altogether, these 337 public TCR sequences were used as reference public gluten-specific TCRs.

Statistical analysis

We analyzed flow cytometry data with FlowJo software (Three Star) and performed statistical analysis with GraphPad Prism software (version 8) (San Diego). The circos plots were generated using the online circos tool (http://circos.ca/circos_online). For statistical analysis of data in Figure 1C (n = 10 untreated CeD patients), 2B (n = 7 untreated CeD patients) and Figure 2E (n = 5 untreated CeD patients) we used paired, two-tailed t-tests. Horizontal bars in Figure 1D, 2B, 2C, 2E, 2F indicate median values.

Study approval

The study was approved by Regional Committee for Medical and Health Research Ethics South-East Norway (project #6544).

Supplementary Figure

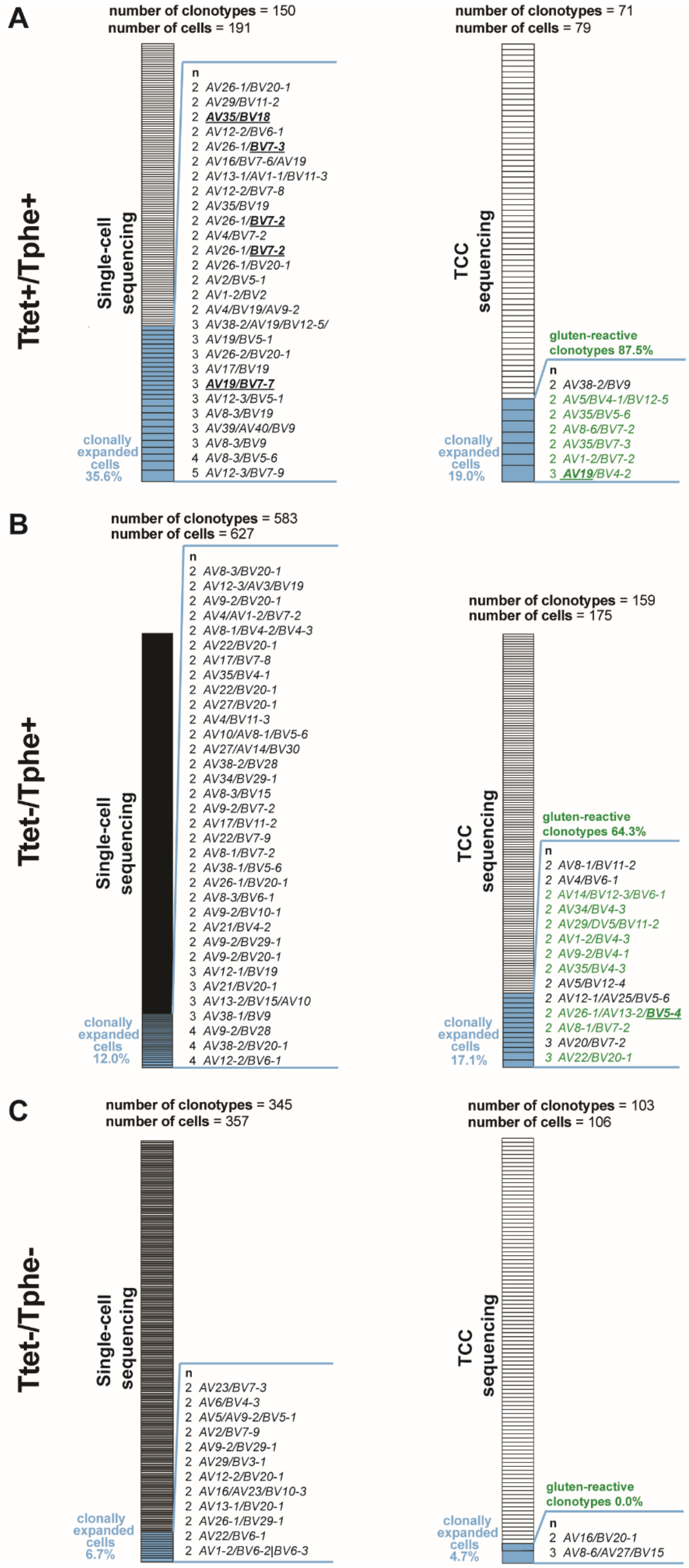

Figure S1. Stack bar representation of clonal distribution. Stack bar representation of clonal distribution among single-cell derived and TCC-derived T cells in each subset (Ttet+/The+, Ttet-/Tphe+ and Ttet-, Tphe-) (n = number of cells per clonotype). A clonotype is identified by paired identical nucleotide TCRαβ chains within a donor. Each box of the stacked bar represents a unique clonotype and the size of the box indicates number of cells per clonotype (i.e. degree of clonal expansion). For expanded clonotypes the *TRAV/TRBV* usage is shown. Boldfaced and underlined V genes indicate clonotypes with the sequences (Vgene-CDR3-Jgene) that have been identified as public TCR sequence of gluten-specific CD4+ T cells.[8, 11] V genes shown in green for TCC indicate those that were reactive to deamidated gluten antigen, and above, the frequency of clonally expanded gluten reactive TCCs. The total frequency of clonally expanded T cells is displayed on the left of each stacked bar graph. The total number of cells and clonotypes for each phenotype are displayed above each stacked bar graph.

Supplementary Tables

Table S1. Antibody panel for ex-vivo cell sorting.

Target	Label	Clone	Producer	Catalog number
ICOS	PerCP/Cy5.5	C398.4A	BioLegend	313518
CXCR3	AF488	G025H7	BioLegend	353710
CD39	PE-Cy7	eBioA1 (A1)	eBioscience (Thermo Fisher Scientific)	25-0399-42
CD4	APC-Cy7	OKT4	BioLegend	317450
CD25	APC-R700	2A3 (RUO)	BD Biosciences	565106
PD-1	PE-CF594	EH12.1 (EH12)	BD Biosciences	565024
CD127	BV711	A019D5	BioLegend	351328
CD11c (Dump)	BV605	3.9	BioLegend	301636
CD14 (Dump)		M5E2	BioLegend	301834
CD19 (Dump)		HIB19 (B4)	BioLegend	302243
CD56 (Dump)		NCAM, Leu-19, NKH1	BioLegend	362537
CD3	BV510	OKT3	BioLegend	317331
CD161	BV421	HP-3G10	BioLegend	339914
TCR specific dominant gluten epitope[a)]	PE	NA	In house production	NA

a) Cocktail of PE-conjugated HLA-DQ2.5:gluten tetramers representing DQ2.5-glia-α1a, DQ2.5-glia-α2, DQ2.5-glia-ω1, DQ2.5-glia-ω2 and DQ2.5-hor3a.

Table S2. Patient information.

Sample ID	Status[a)]	HLA type	Marsh score	Anti-TG2[b)]	Anti-DGP[c)]	Generation of TCCs[d)]	TCR[e)] sequencing of the TCCs[d)]	Single-cell TCR[e)] sequencing from SCS[f)]
CD5030	UCeD	DQ2.5	3A	9	6.2	Yes	Yes	No
CD5037	UCeD	DQ2.5	3B/C	21	2.3	Yes	Yes	Yes
CD2135	UCeD	DQ2.5	3B	40	76	Yes	Yes	Yes
CD2126	UCeD	DQ2.5	3B	70.5	99	Yes	Yes	Yes
CD5063	UCeD	DQ2.5	3C	75	16	Yes	Yes	No
CD2219	UCeD	DQ2.5	3B	57.7	55	No	NA	Yes
CD2326	UCeD	DQ2.5	3B	100	100	No	NA	Yes
CD5028	UCeD	DQ2.5	3C	11	10	No	NA	Yes
CD5060	UCeD	DQ2.5	3C	>128	89	No	NA	Yes
CD5058	UCeD	DQ2.5	3B/C	33	19	No	NA	Yes
CD5065	UCeD	DQ2.5	3B	65	13	No	NA	Yes
CD5006	UCeD	DQ2.5	3B/C	>128	55	No	NA	No
CD5007	UCeD	DQ2.5	3A/B	67	20	No	NA	No
CD5044	UCeD	DQ2.5	3A/B	76	30	No	NA	No
CD5038	UCeD	DQ2.5	3B/C	16	8.9	Yes	Yes	No

a) All samples were obtained from Untreated Celiac Disease patients (UCeD)
b) TG2, Immunoglobulin A-anti-Transglutaminase 2, reference value < 4, [AU], arbitrary units
c) DGP, Immunoglobulin G-anti-Deamidated Gliadin Peptide, Reference value < 20 [AU]
d) T cell clone (TCC)
e) T cell receptor (TCR)
f) Single-cell suspension (SCS)

Table S3. Antibody panel for re-staining of TCCs (used in Figure 2C).

Target	Label	Clone	Producer	Catalog number
Complex of gluten T-cell epitope and HLA-DQ2.5[a)]	PE	NA	In house production	NA
CLIP2[b)]	APC	NA	In house production	NA
CD4	APC-Cy7	OKT4	BioLegend	317450
CD8	PerCP	SK1	BioLegend	344708
Dead cells	Aqua		Invitrogen	L34957

a) Cocktail of PE-conjugated HLA-DQ2.5:gluten tetramers representing the gluten T-cell epitopes DQ2.5-glia-α1a, DQ2.5-glia-α2, DQ2.5-glia-ω1, DQ2.5-glia-ω2 and DQ2.5-hor3a.

b) APC-conjugated DQ2.5:CLIP2 tetramer to exclude unspecific tetramer binding.

Table S4. Use of public TCRs[a)] by clonally expanded T cells identified within the Ttet+/Tphe+ and Ttet-/Tphe+ subset of T cells.

Phenotype	Patient ID	TRA	TRB	Number of cells	TCC name (Gluten reactivity)
Tet+Tphe+	CD5065	***AV19_ALSEAGANSKLT_AJ56***[c)]	***BV7-7_ASSLLAGGDTQY_BJ2-3***[c)]	3	NA
	CD2326	***AV35_AGQLDSGTYKYI_AJ40***[c)]	***BV18_ASSPAGWDTEAF_BJ1-1***[c)]	2	NA
	CD5037	**AV19_ALSEAGYGGATNKLI_AJ32**[b)]	BV4-2_ASSRTSENTGELF_BJ2-2	3	5037.1.6 (R), 5037.1.8 (R) & 5037.1.13 (R)
	CD5037	AV13-1_AASINNNARLM_AJ31 |**AV26-1_IVYNTDKLI_AJ34**[b)]	**BV7-3_ASSIRSTDTQY_BJ2-3**[b)]	2	NA & 5037.1.7(NR)
	CD5065	AV26-1_ISNYGGSQGNLI_AJ42	**BV7-2_ASSLRYTDTQY_BJ2-3**[b)]	2	NA
	CD2326	AV26-1_IGGGGNKLT_AJ10	**BV7-3_ASSIRSTDTQY_BJ2-3**[b)]	2	NA
	CD5065	AV26-1_IADYGGSQGNLI_AJ42	**BV7-2_ASSLRYTDTQY_BJ2-3**[b)]	2	NA
Tet-Tphe+	CD2135	AV13-2_ASNNRKLI_AJ38 | AV26-1_IVSSRTGGFKTI_AJ9	**BV5-4_ASSLAQGGDTQY_BJ2-3**[b)]	2	2135.2.35 (R) & 2135.2.4 (R)

a) Public TCR - TCRα, TCRβ and paired TCRαβ sequences of the expanded clonotypes within the Ttet+/Tphe+ and Ttet-/Tphe+ subset were analyzed for the presence of public gluten-specific TCRs identified in previous publications.[8]
b) The public TCRα and TCRβ sequences are **boldfaced**
c) the paired public TCRαβ sequences are **boldfaced** and *italicized*
d) NA, not available - when the data was derived from single cell TCR sequencing of directly sorted T cells
e) R; reactive or NR; not reactive – when the data was derived from TCR sequencing the gluten reactivity is known

References

[1] J. F. Ludvigsson, D. A. Leffler, J. C. Bai, F. Biagi, A. Fasano, P. H. Green, M. Hadjivassiliou, K. Kaukinen, C. P. Kelly, J. N. Leonard, K. E. Lundin, J. A. Murray, D. S. Sanders, M. M. Walker, F. Zingone, C. Ciacci, *Gut.* **2013**, *62*, 43.

[2] L. F. Risnes, A. Christophersen, S. Dahal-Koirala, R. S. Neumann, G. K. Sandve, V. K. Sarna, K. E. Lundin, S. W. Qiao, L. M. Sollid, *J Clin Invest.* **2018**, *128*, 2642.

[3] H. Quarsten, S. N. McAdam, T. Jensen, H. Arentz-Hansen, Ø. Molberg, K. E. Lundin, L. M. Sollid, *J Immunol.* **2001**, *167*, 4861.

[4] J. A. Tye-Din, J. A. Stewart, J. A. Dromey, T. Beissbarth, D. A. van Heel, A. Tatham, K. Henderson, S. I. Mannering, C. Gianfrani, D. P. Jewell, A. V. Hill, J. McCluskey, J. Rossjohn, R. P. Anderson, *Sci Transl Med.* **2010**, *2*, 41ra51.

[5] L. M. Sollid, J. A. Tye-Din, S. W. Qiao, R. P. Anderson, C. Gianfrani, F. Koning, *Immunogenetics.* **2020**, *72*, 85.

[6] M. Raki, L. E. Fallang, M. Brottveit, E. Bergseng, H. Quarsten, K. E. Lundin, L. M. Sollid, *Proc Natl Acad Sci U S A.* **2007**, *104*, 2831.

[7] Ø. Molberg, K. Kett, H. Scott, E. Thorsby, L. M. Sollid, K. E. Lundin, *Scand J Immunol.* **1997**, *46*, 103.

[8] S. Dahal-Koirala, L. F. Risnes, R. S. Neumann, A. Christophersen, K. E. A. Lundin, G. K. Sandve, S. W. Qiao, L. M. Sollid, *Front Immunol.* **2021**, *12*, 639672.

[9] E. Alamyar, P. Duroux, M. P. Lefranc, V. Giudicelli, *Methods Mol Biol.* **2012**, *882*, 569.

[10] D. A. Bolotin, S. Poslavsky, I. Mitrophanov, M. Shugay, I. Z. Mamedov, E. V. Putintseva, D. M. Chudakov, *Nat Methods.* **2015**, *12*, 380.

[11] S. Dahal-Koirala, R. S. Neumann, J. Jahnsen, K. E. A. Lundin, L. M. Sollid, *Eur J Immunol.* **2020**, *50*, 256.
